# Prevalence of ADHD Among Students of Zahedan University of Medical Science in Iran

**Published:** 2013

**Authors:** Monire Mosalanejad, Leili Mosalanejad, Kobra Lashkarpour

**Affiliations:** 1 General Practitioner, Zahedan University of Medical Sciences, Zahedan, Iran.; 2 Assistant Professor, Department of Mental Health, School of Nursing and Paramedical, Jahrom University of Medical Sciences, Jahrom, Iran.; 3 Assistant Professor, Department of Psychiatry, Zahedan University of Medical Sciences, Zahedan, Iran.

**Keywords:** ADHD, Adult ADHD, Conners’ Adult ADHD Rating Scale, Student

## Abstract

**Objective:** Attention-deficit/hyperactivity disorder (ADHD) is a common mental disorder in adults that was under-diagnosed until recently. Due to probable consequences of ADHD such as occupational and educational dysfunctions and substance use, this disorder is becoming more and more of a concern. This study aimed to investigate ADHD symptoms among students of Zahedan University of medical sciences, Iran.

**Methods:** This cross-sectional study was performed in Zahedan University of Medical Sciences from 2008 to 2009. Our sample included 1500 individuals who were chosen using simple sampling method. Considering the goal of the investigation, two questionnaires were distributed among students including demographic information form and the Conners' Adult ADHD Rating Scales–Self Report (Screening Version, CAARS-S:SV). Data analysis was done using descriptive and analytical statistics in SPSS software.

**Results: **Out of 1500 questionnaires distributed among students, 913 were completed. 589 students (64.5%) were female and 324 (35.5%) were male. The Mean age of participants was 21.7 ± 3.2 years. ADHD symptoms were defined based on the Conner’s adult test. Based on CAARS-S: SV, inattention/memory, hyperactivity/restlessness, impulsiveness/emotional lability, and problems with self-concept subscale symptoms were found in 107 (11.7%), 109 (12%), 121 (13.2%), and 30 (3.3%) respondents, respectively. These findings were significantly higher than average.

**Conclusions:** According to our results, it seems that the prevalence of ADHD is high among students. Thus, more screening is required in this population in order to diagnose and treat the disorder earlier and prevent its consequences, such as substance abuse.

**Declaration of interest:** None.

## Introduction

Attention deficit hyperactivity disorder (ADHD) is a developmental-nervous-behavioral disorder mostly diagnosed in childhood. Its prominent features are attention deficit, hyperactivity, and impulsiveness, which can cause poor occupational, academic, and social performance in the future (1). The prevalence of ADHD ranges from 2% to 22%. Based on the specifications mentioned in the Diagnostic and Statistical Manual of Mental Disorders, Fourth Edition (DSM-IV), the prevalence of ADHD was reported to be more than 9.5% in the US. Moreover, it was different in respect to gender; it was more frequent among boys than girls 2:1 to 9:1 (1-6). 

ADHD is a mental disorder in adults and in spite of many published studies from mid-1970 until recently, it was rarely diagnosed in adults. About 30-70% of children who suffer from ADHD will suffer from the same disorder during puberty, so that the physicians prescribe ongoing treatment for them (7, 8).

This disorder was described previously as a disorder pertaining to children and youth, but Cohort studies following up on children with ADHD showed that the disease conditions continue noticeably until adulthood depending on the disease intensity. Studies have shown poor mental results for patients in their adulthood, sometimes with high percentage of educational deficiency, drug abuse, personality disorder, and etcetera (9, 10).

ADHD is prevalent among 30-50% of patients with psychiatric problems suffering from drug abuse (9, 11-13).

According to the results of a study carried out in Iran in 2004 on 409 students the prevalence of disorders due to hyperactivity-attention deficit was reported to be 3.7% (8).

Characteristic clinical features of ADHD in adults include hyperactivity, attention deficit, intense emotional response, low provocation threshold, sudden and intense anger, impulsiveness, instability in interpersonal relations, occupational and educational frustration, and alcohol abuse (2). Attention disorder is problematic in adults especially in college students. Adults suffering from ADHD have problems in organizing, completing their duties, and doing them on time, listening to others’ words, making decisions, and reading. They may also suffer from metacognitive deficit (14).

The incidence of ADHD in adults is unclear, but it is more than what was imagined before. It is increasingly diagnosed nowadays, and still requires more investigation. Other symptoms in adults include impulsiveness and attention deficit. Failure to diagnose and/or treat this disorder is accompanied by many complications, such as secondary depression and low self-esteem (due to functional disorders), which can influence occupational and social functions. Pharmacotherapy includes amphetamine (5-60 mg daily) or methylphenidate (5-60 mg daily) (15, 16). Simultaneous occurrence of ADHD and drug dependence has drawn more attention to this issue in recent clinical experiments. The positive response to treatment includes higher attention rate, higher impulse control, and improved mood. Perhaps it would be necessary to continue psychiatric treatments ceaselessly. As these medications have potential to be abused, clinicians should monitor the patient’s responses to such medications (17). 

These two disorders are interrelated in some ways. Patients suffering from ADHD may begin to abuse drugs at an early age and show a more frequent intense model of abuse (18).

Researchers using factor analysis have consistently found support for an inattention factor both in children and adults. Findings have been mixed regarding whether hyperactivity and impulsiveness reflect one or two dimensions (19). ADHD patients may not succeed in their efforts to qualify for a job (20).

These patients may need to select some special types of occupation to enable them to adapt continuously with previous conditions. They usually have many problems with their colleagues and employers such as being late, absent, and careless (21).

Other findings support the results that ADHD is associated with greater disruptive behaviors, substance abuse, mood and anxiety disorders, early onset of major depression, dysthymia, oppositional defiant disorder, and conduct disorder. Males had a higher risk of disruptive behavior disorders. Females with oppositional defiant disorder also have a high risk of depression and anxiety (22). 

According to previous studies, ADHD control is important in order to prevent one of its most dangerous consequences; drug abuse (23). ADHD youth are at high risk of a wide range of adverse psychiatric outcomes including markedly elevated rates of antisocial, addictive, mood, and anxiety disorders. These prospective findings provide further evidence for the high morbidity associated with ADHD across the life-cycle, and emphasize the importance of early diagnosis of this disorder for prevention and intervention strategies (24).

Due to what was stated about this disorder and future social, familial, and educational damages, and the limited number of studies about adults' ADHD, we decided to do this study. The aim of this study was to determine the prevalence of ADHD in students.

## Materials and Methods

This descriptive study included all students of Zahedan University of Medical Sciences, Zahedan (in the southeast of Iran). Two questionnaires were used; one included demographic, educational, and medical history, and the other one was the Conners' Adult ADHD Rating Scales–Self Report: Screening Version (CAARS-S:SV) (25). The questionnaires were anonymous and self-reported questionnaires. 

All students were assured regarding the confidentiality of their information. Students were asked to fill out the questionnaires at the beginning of their classes. Comprehensive information and enough time were provided by researchers. All questionnaires were collected by researchers after being answered by the students. It should be noted that the CAARS-S:SV is applied to screen adults for ADHD, and clinical interview is necessary in order to confirm the diagnosis of this disorder.

CAARS-S:SV has an approved reliability and validity. It includes 30 items with 0 to 3 points which can assess the following four subscales (26): 

A: Inattention/memory problem

B: Restlessness/hyperactivity

C: Emotional instability/impulsiveness 

D: Problems concerning general self-image 

This scale has been normalized in the Iranian population by Arabgol et al. on 20 students of Shahid Beheshti University of Medical Sciences, Iran, and its content validity and reliability has been reported by Chronbach's alpha (0.81) (6). 

The raw score of each subscale was changed to T-scores by appropriate norm tables (T-scores had the mean (± standard deviation) of 50 (± 10)). Scores higher than 65 were considered as clinically significant; therefore, T-scores of 66-77 were higher than the mean. Scores over 70 were much higher than the mean. Furthermore, scores higher than 80 indicated sever pathological disorders and exaggerated bad symptoms (8).

Data was analyzed using descriptive statistics for distribution of data as mean and standard deviation, and analytical statistics as student t-test to compare the mean of CAARS-S:SV scores and its subscales among men and women by SPSS for Windows (version 13; SPSS Inc., Chicago, IL., USA). Statistical significance was evaluated with an alpha level of 0.05.

## Results

Of 1,700 students in all fields of study 913 students answered the questionnaires completely. 589 respondents (64.5%) were female and 324 (35.5%) were male. They were 18-44 years old with the mean age of 21.7 (± 3.2). 

Based on CAARS-S:SV, inattention/memory, hyperactivity/restlessness, impulsiveness/emotional lability, and problems with self-concept subscale symptoms were found in 107 (11.7%), 109 (12%), 121 (13.2%), and 30 (3.3%) respondents, respectively. They were significantly higher than average.

To determine the severity of the disease, the raw score of each subscale was changed to T-scores by appropriate norm tables. As is shown in table 1, the rate of ADHD symptoms in impulsiveness/emotional lability, and hyperactivity/restlessness subscale scores were significantly higher compared to other subscales. 

**Table 1:** The severity of attention-deficit/hyperactivity disorder (ADHD) symptoms on the basis of standard indexes in students

	**Higher than mean ** **T = 66-70**	**Much higher than** **mean** **T > 70**
**Inattention**	47 (5.1%)	60 (6.6%)
**Hyperactivity**	51 (5.6%)	58 (6.4%)
**Impulsiveness**	36 (3.9%)	85 (9.3%)
**Self-concept**	19 (2.1%)	11 (1.2%)

Regarding the frequency of ADHD symptoms inattention/memory problems, hyperactivity/restlessness, impulsivity/emotional lability, and problems in self-concept subscale scores were higher or much higher than the mean in both sexes (Table 2).

Most of the respondents (25.7%) were paramedical students. ADHD symptoms in A, B, C, and D subscales are given in table 3 according to their majors. There were significant percentages of ADHD subscales in the range of higher than mean and much higher than mean.

Students' T-scores average of the respondents regarding inattention, hyperactivity, impulsiveness, and self-concept disorder subscales based on their gender showed that the mean in males was higher than that in females in all subscales except for self-concept disorder. According to these results a significant difference was observed between the inattention, hyperactivity, and impulsiveness scores of male and female respondents (p ≤ 0.001), but not in the self-concept score (p = 0.679) (Table 4).

## Discussion

Adult attention-deficit/hyperactivity disorder (ADHD) is a mental health condition exhibited by the difficulty to maintain attention, hyperactivity, and impulsive behavior (1, 8). 

In our investigation, 15.4% of the respondents had ADHD symptoms. It was reported as 0.3 to 6% in other studies (1, 8, 9). Fernandez has done the same study on 374 students in Baliwasan Central School in Zamboanga and has reported an average prevalence of 5.3%. He has estimated ADHD prevalence rate to be between 3-7% (27).

Fayyad et al. administered a similar study in ten countries in the Americas, Europe, and the Middle East on 11422 respondents aged 18-44 years. Adult ADHD average prevalence was reported to be 3.4% (1.2–7.3%). This research showed that socio-economic status influences the prevalence of this disorder. In this cross-national study, different prevalence rates were reported in low-income countries (1.9%) compared with high-income countries (4.2%) (28).

**Table 2:** Distribution of the severity of attention-deficit/hyperactivity disorder (ADHD) symptom based on gender

**Gender**	**Subscales**	**Normal range** **n (%)**		**Higher than mean** **n (%)**		**Much higher than mean** **n (%)**	
**Male**	Inattention/Memory problems	260	(80.2)	28	(8.6)	36	(11.1)
	Hyperactivity/Restlessness	265	(81.8)	24	(7.4)	35	(10.8)
	Impulsivity/Emotional lability	256	(79)	18	(5.6)	50	(15.4)
	Problems with self-concept	316	(97.5)	3	(0.9)	5	(1.5)
**Female**	Inattention/Memory problems	546	(92.7)	19	(3.2)	24	(4.1)
	Hyperactivity/Restlessness	265	(81.8)	27	(4.6)	23	(3.9)
	Impulsivity/Emotional lability	256	(79.0)	18	(5.6)	50	(15.4)
	Problems with self-concept	567	(96.3)	16	(2.7)	6	(1.0)

**Table 3:** The distribution of attention-deficit/hyperactivity disorder (ADHD) indexes on the basis of their major

**ADHD subscale**	**Major**	**Normal**		**higher than mean**		**Much higher than mean**	
		**Rate**	**Percentage**	**Rate**	**Percentage**	**Rate**	**Percentage**
**Inattention**	Medicine	198	88.8	13	5.8	12	5.4
	Paramedical	202	86.0	11	4.7	22	9.4
	Dentistry	97	87.4	5	4.5	9	8.1
	Nursing	92	90.2	5	4.9	5	4.9
	Midwifery	80	98.8	1	1.2		
	Health	136	95.5	11	69.0	12	7.5
	Unknown	2	10.0				
**Hyperactivity**	Medicine	202	90.6	12	5.4	9	4.0
	Paramedical	204	86.8	13	5.5	18	7.7
	Dentistry	97	87.4	5	4.5	9	8.1
	Nursing	92	90.2	4	3.9	6	5.9
	Midwifery	77	95.1	2	2.5	2	2.5
	Health	131	82.4	14	8.8	14	8.8
	Unknown	2	10.0				
**Impulsive**	Medicine	201	90.1	5	2.2	17	7.6
	Paramedical	195	83.0	14	6.0	26	11.1
	Dentistry	95	85.6	5	4.5	11	9.9
	Nursing	90	88.2	3	2.9	9	8.8
	Midwifery	79	97.5	2	2.5	2	2.5
	Health	131	82.4	9	5.7	19	11.9
	Unknown	2	10.0				
**Self-concept disorder**	Medicine	216	96.9	4	1.8	3	1.3
	Paramedical	227	96.6	5	2.1	3	1.3
	Dentistry	105	64.6	4	3.6	2	1.8
	Nursing	100	98.0	1	1.0	1	1.0
	Midwifery	80	98.0	1	1.2		
	Health	153	96.2	4	2.5	6	1.3
	Unknown	2	10.0				

**Table 4:** Differences in the attention-deficit/hyperactivity disorder (ADHD) symptoms on the basis of gender

	**Self-concept disorder**	**Impulsiveness**	**Hyperactivity**	**Inattention**
	**Mean (± SD)**	**Mean (± SD)**	**Mean (± SD)**	**Mean (± SD)**
**Male**	45.04 (8.88)	54.47 (13.79)	51.45 (12.93)	55.01 (12.54)
**Female**	49.07 (8.45)	50.69 (10.19)	48.40 (11.00)	51.88 (9.39)
**T**	0.414	5.24*	4.61*	5.39*

* P < 0.05

Other studies have shown different results according to different methodological methods and estimated a prevalence ranging from 3.7% to 8.9% (29). These results are closer to the current study.

Finally, Polanczyk et al. have undertaken a systematic review and stated that the worldwide prevalence of ADHD was 5.29%. This suggests that geographic conditions play a limited role in ADHD/HD prevalence estimates worldwide (30).

Some evidences demonstrate that cultural and contextual aspects (psychosocial adversity) are predisposing risk factors (31). In other research the symptoms reported did not vary significantly by gender (32).

The prevalence of ADHD in our study was higher than that in other recent studies (28-31). This difference could be due to social and cultural factors (28, 31, 33).

It is also noteworthy that 557 participants were eliminated from our study due to not completing the questionnaires. They might have also been suffering from ADHD disorder. Therefore, a higher prevalence rate was also possible among students. These symptoms can disturb students’ academic efficiency, lengthen their study, and even disturb their development (6, 2). ADHD is associated with lower levels of education and employment status (30).

In addition, impulsiveness can disturb interpersonal and marital relationships, and occupational functions (31).

In this study, 21% of participants of both sexes had impulsiveness index higher or much higher than mean. Few studies have investigated the ADHD adulthood prevalence rate and its consequences in Iran. In a study performed in Tehran, 4.9% of the study participants had impulsiveness index higher or much higher than mean (6). The lowest percentage related to ADHD symptoms (0.9%) with an intensity of higher than the mean was related to self-concept in the male group. This study confirms our results. 

Another study showed that the maximum rate was related to subscale B (7.8% for hyperactivity-restlessness) and the minimum rate (2%) was related to subscales D (problems concerning general self-concept) and A (Low attention–memory) (27). However, our results are not in line with these results.

As various researches have shown, rate of ADHD symptoms in different countries is varied due to geographic aria, race, and psychosocial adversity. Moreover, its rate is believed to be higher in high-income countries; these cases were not examined in the present study.

Recent studies have also showed a significant difference between the male and female inattention, hyperactivity, and impulsiveness symptoms (p ≤ 0.001), but not self-concept. DuPaul et al. have performed a cross-gender and cross-national prevalence study among university students which did not find a significant difference between the two sexes (32). These results are not in accordance with the current study. This can be due to the difference between gender roles and socio-cultural factors in different communities (28, 33). Moreover, there is now clear evidence that neurobiological and environmental factors contribute to these phenotypes. A mounting body of evidence also suggests the interactive effects of genetic and environmental risks (34). 

According to another study performed in Tehran the maximum and minimum ADHD symptoms were related to the impulsiveness subscale (13.2%) and self-concept subscale (3.3%), respectively. According to the study performed by Arabgol et al., the maximum and minimum rates were related to hyperactivity (7.8%) and self-concept problem subscales (2%), respectively. These results confirm our results. Furthermore, symptoms intensity in subscales A, B, and C were mostly related to the intensity much higher than mean. Symptoms intensity percentage was higher than mean only in self-concept problems (6).

Another study about ADHD symptoms on 1,209 university students from three countries (Italy, New Zealand, and the United States) reported that inattention and hyperactivity-impulsiveness among Italian students were significantly higher than that in the students from the United States. However, inattention symptoms in the students of New Zealand were reported to be higher than that in the students from the United States (32).

Some studies have tried to address the consequences of this disorder and the importance of early diagnosis. This disorder is associated with a higher risk of antisocial personality (10 times more than the control group) and substance and alcohol abuse (4-5 times more than the control group). Adults’ ADHD increases mood disorders (2-6 times), anxiety (2-4 times), communication disorders (2 times), and learning disorders compared with the control group (35).

Other researches have confirmed the above results and emphasized that ADHD, alone and in combination with other psychiatric problems, is a risk factor for the development of substance use disorder (SUDs) in youths. These studies reported that approximately one fifth of adults with SUDs have ADHD (36-38). 

People with ADHD symptoms, particularly attention deficit disorders, have difficulty in communication and conversation (39). In addition, many of these patients suffer from learning impairments (30). 

## Conclusion

Due to the high prevalence of ADHD in adults and the importance of its early diagnosis and treatment, there is a need for more frequent screening.

On the other hand, applied approaches such as students’ psychological assessment during primary enrollment, informing counselors and paying more attention to this disorder in university counseling centers, on time referral of patients to psychiatrists, and finding patients are necessary in order to decrease the unfavorable effects of ADHD on their occupational, educational, and familial lives.
